# Supplementary material for: Piwi–piRNA complexes induce stepwise changes in nuclear architecture at target loci
Source: EMBO J. 2021 Aug 2;40(18):e108345. doi: 10.15252/embj.2021108345 (PMC8441340; doi:10.15252/embj.2021108345)
Supplement: Supplementary file 2 — Expanded View Figures PDF [file EMBJ-40-e108345-s002.pdf]

Expanded View Figures

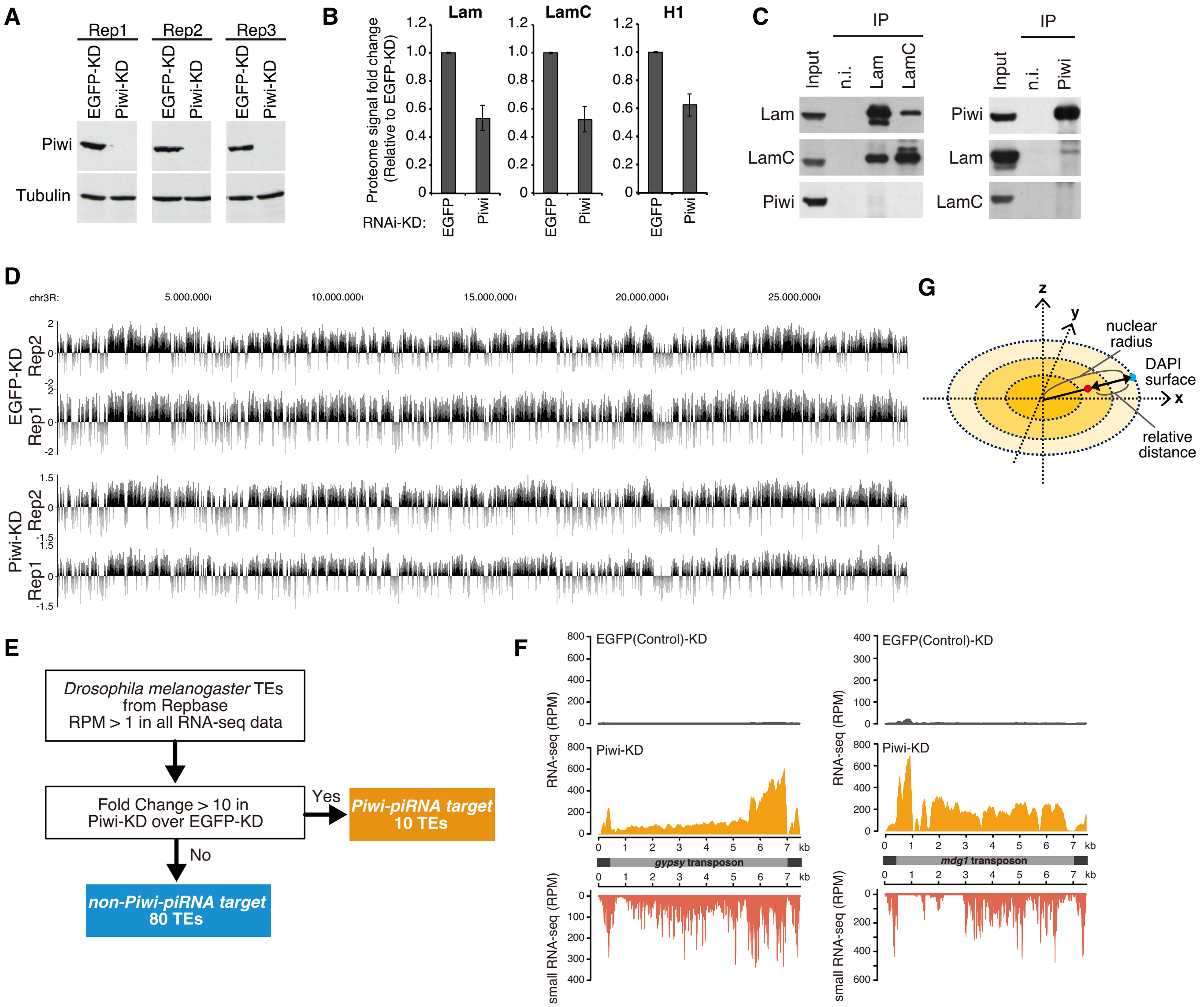

Figure EV1. Piwi–piRNA target TEs are tethered to the nuclear periphery.

- A RNAi KD of Piwi protein in three replicates, followed by Western blotting (WB). All the replicates show significantly decreased Piwi protein levels.
- B Fold change in extracted ChEP signals for Lam, LamC, and H1 (positive control). Error bars indicate SD ($n = 3$), adjusted P-values < 0.05. Significant decreases in ChEP signals were observed for these proteins.
- C Immunoprecipitation (IP) from OSC lysate using anti-Lam, LamC, or Piwi antibody, followed by WB using indicated antibodies. Mouse immunoglobulin G (n.i.) was used for control IP. Association between Lam and Piwi could not be detected.
- D Chromosome-wide DamID-seq read distribution of Chr3R, showing that replicates have highly similar results. Replicates are therefore merged for further analysis.
- E Of *Drosophila melanogaster* TEs registered in Repbase, those with RPM values > 1 in all RNA-seq data were extracted. Among those, 10 TEs with > 10-fold change in Piwi-KD/EGFP-KD expression were categorized as "piRNA target TEs". The other 80 TEs were grouped as "non-piRNA target TEs".
- F Density plots for RNA-seq signals and small RNA-seq signals over consensus sequences from piRNA target TEs (*gypsy* and *mdg1*) in EGFP (control)-KD or Piwi-KD cells. piRNA target TEs are de-repressed upon Piwi-KD, and small RNAs are mapped to their antisense direction.
- G Diagram describing the measurement of oligo-FISH signal from the nuclear periphery. DAPI surface was used to define nuclear periphery. The ratio of the distance from the FISH signal to the DAPI surface against the distance from the DAPI center to the DAPI surface.

© 2021 The Authors

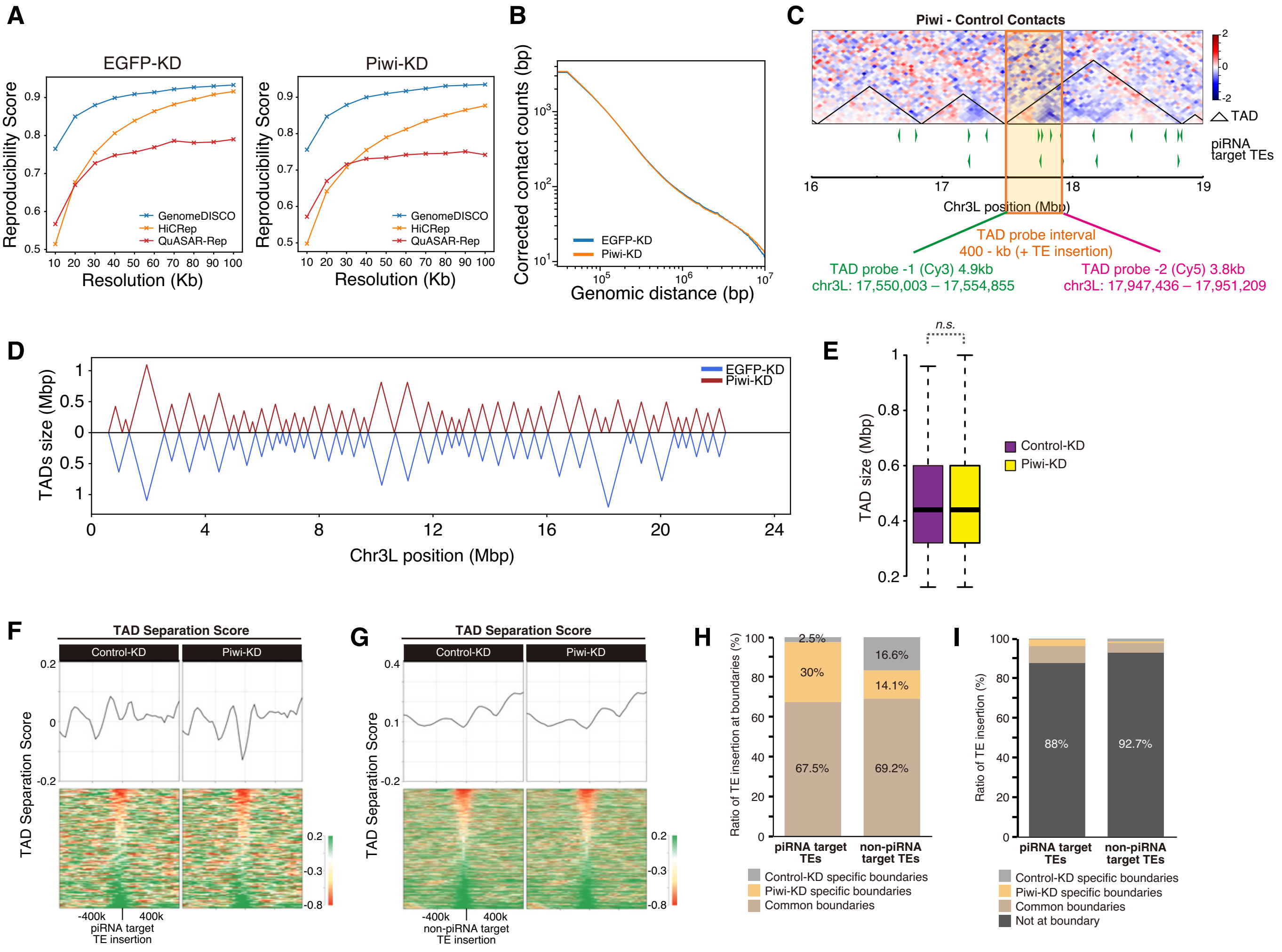

Figure EV2. Changes in chromatin conformation upon Piwi–depletion.

A Evaluation of replicate Hi-C matrix at indicated resolutions. Three different softwares were used to calculate the reproducibility score at each range of resolutions. This analysis indicates that the optimal and stable resolution for the datasets (EGFP-KD and Piwi-KD) is 40K.

B Relative contact probability (RCP) plot showing corrected contact counts at different genomic ranges, genome widely. RCP values are compared between EGFP (control)-KD (blue) and Piwi-KD (orange) OSCs.

C Diagram describing the probe design for oligo-FISH detection of intra-TAD interaction. Differential interaction heatmap for 16–19 Mbp of chr3L shown in $\log_2$ fold change. Two probes (TAD probe-1 and TAD probe-2) were designed within the same TAD but apart in the distance about 400 kb (plus TE insertion), and the distance between two TAD probes was measured in order to visualize the changes in intra-TAD interactions.

D TADs calculated using EGFP (control)-KD (blue) or Piwi-KD (red) Hi-C contacts, for chromosome 3L. Most TADs are common to both EGFP and Piwi-KD, but there are some which have shifted TAD boundaries.

E Boxplots showing the size of the TADs in EGFP (control)- or Piwi-KD OSCs based on the Hi-C contact dataset. Boxplot whiskers, box, and central band show 1.5 times the inter-quartile range, the first to the third quartile, and median, respectively (n = 228 for Control-KD, n = 220 for Piwi-KD). P-values were calculated using the Wilcoxon rank-sum test.

F Average TAD separation score at piRNA target TE insertions for Control (EGFP, left)- or Piwi (right)-KD (upper). Heatmap showing changes in the TAD separation score for each piRNA target TE insertion sites (lower). This analysis, together with control analysis data shown in Fig EV2G, indicates a decrease in separation score upon Piwi-KD occurs specifically at piRNA target TE insertion sites.

G Average TAD separation score at non-piRNA target TE insertions for Control (EGFP, left)- or Piwi (right)-KD (upper). Heatmap showing changes in the TAD separation score for each piRNA target TE insertion sites (lower).

H Stacked bar chart showing the ratio of TE insertion at each type of TAD boundary. Boundaries found only in Control (EGFP)-KD OSCs, only in Piwi-KD OSCs, and those common to both KD samples are shown by ratio. This shows that Piwi-KD specific boundaries are enriched with piRNA target TE insertions.

I Stacked bar chart showing the ratio of TE insertion at each defined genomic region. Boundaries that are found only in Control (EGFP)-KD OSCs, only in Piwi-KD OSCs, and those common to both KD samples are shown together with the ratio of TE insertions which are not located at the boundary. This shows that most of the TE insertions are not located at the TAD boundaries, but rather within TADs.

© 2021 The Authors

Figure EV3. Changes in chromatin state upon Piwi–depletion.

A Density plots for normalized H3K9me3, H3K27Ac, H3K4me3, and H1 ChIP-seq signals over consensus sequences of *gypsy*, *mdg1*, and *roo* TEs in EGFP-KD (control) or Piwi-KD cells. ChIP signal depths on EGFP (gray)- and Piwi (red)-KD samples are shown.

B Metaplots showing H3K9me3, H3K27Ac, H3K4me3, and H1 ChIP-seq signals for genomic regions around euchromatin insertions of *gypsy*, *mdg1*, and *roo* TEs. Fold change in Piwi-KD compared with EGFP (control)-KD is shown for indicated ChIP (red) and input (gray) signals. The *y*-axis is a $\log_2$ scale.

C Diagram showing the boxB-λN tethering system. λN-Nxf2 is recruited to the nascent transcript of luciferase reporter via its 14× boxB. The approximate region targeted for ChIP-qPCR is indicated.

 © 2021 The Authors

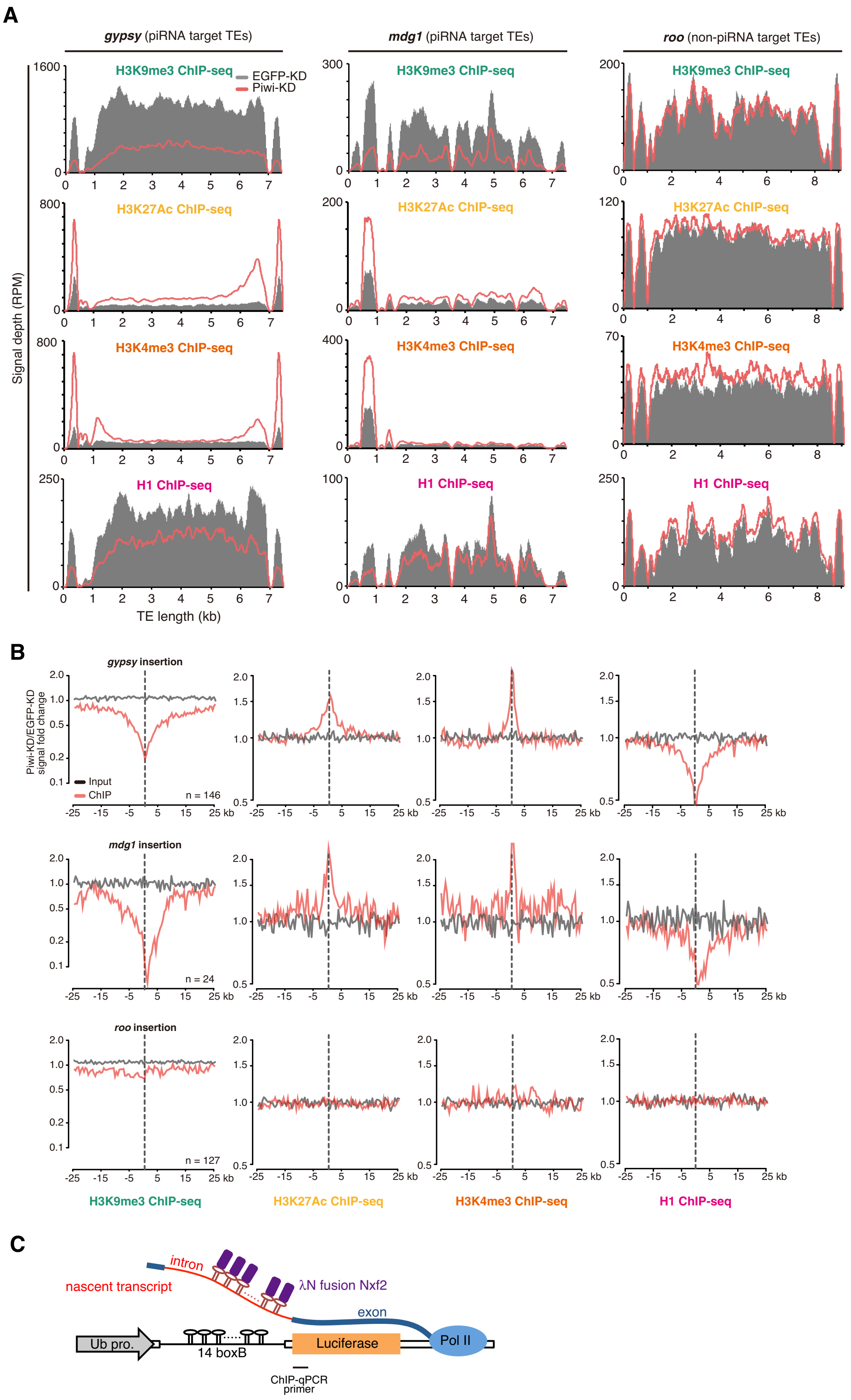
A
gypsy (piRNA target TEs)
mdg1 (piRNA target TEs)
roo (non-piRNA target TEs)
H3K9me3 ChIP-seq
EGFP-KD
Piwi-KD
H3K27Ac ChIP-seq
H3K4me3 ChIP-seq
H1 ChIP-seq
Signal depth (RPM)
TE length (kb)
B
gypsy insertion
mdg1 insertion
roo insertion
Piwi-KD/EGFP-KD signal fold change
Input
ChIP
n = 146
n = 24
n = 127
H3K9me3 ChIP-seq
H3K27Ac ChIP-seq
H3K4me3 ChIP-seq
H1 ChIP-seq
C
intron
nascent transcript
λN fusion Nxf2
exon
Pol II
Ub pro.
14 boxB
Luciferase
ChIP-qPCR primer

Figure EV3.

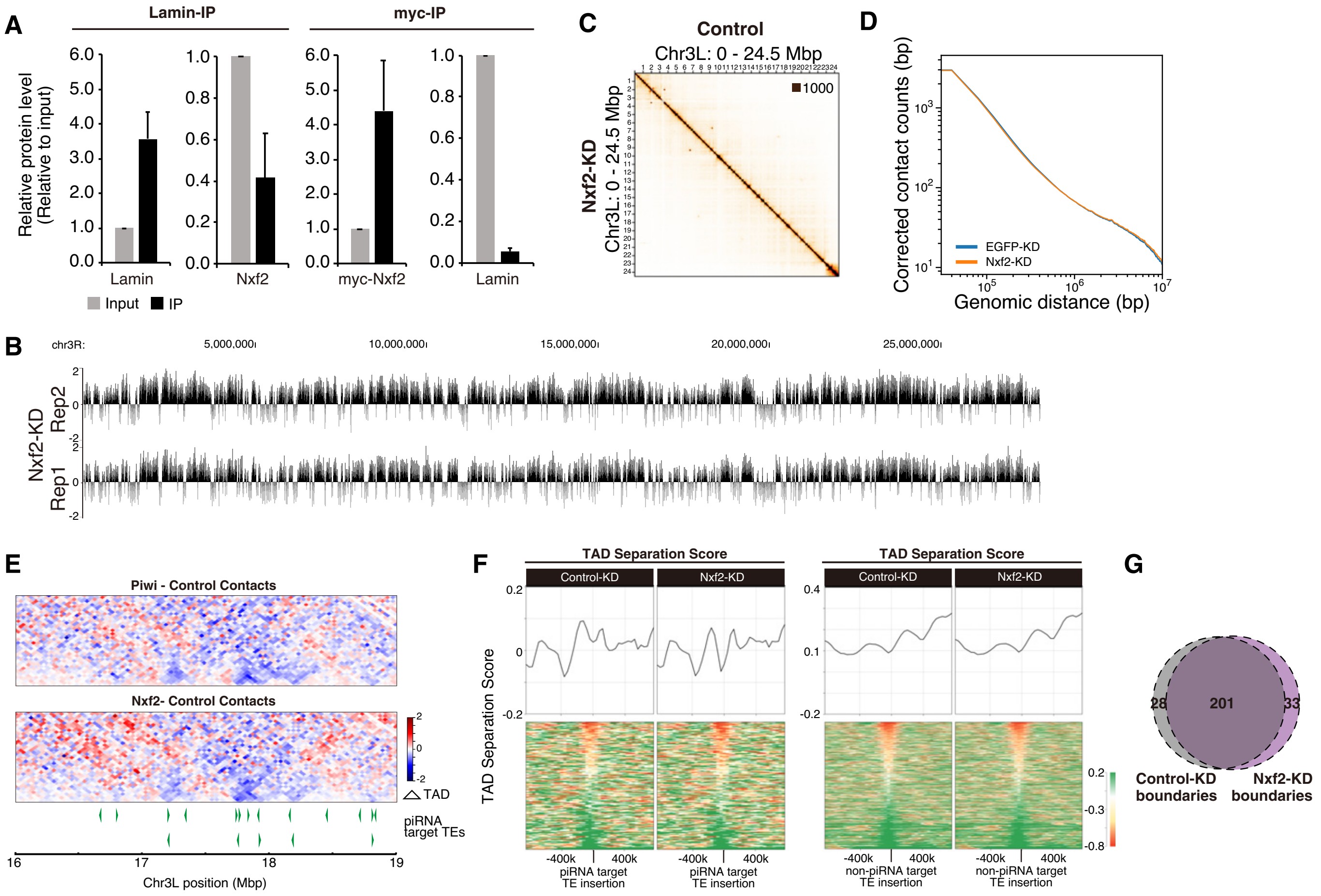

Figure EV4. Nxf2 regulates nuclear localization and chromatin conformation.

A Immunoprecipitation followed by Western blots shown in Fig 5A has been performed in three replicates, and bands were quantified. Error bars indicate SD. The Western blot analysis was reproducible.

B DamID-seq read distribution of Chr3R chromosome-wide, showing that replicates have highly similar results. Replicates are therefore merged for further analysis.

C Hi-C interaction matrices of the EGFP-KD (control) and Nxf2-KD OSCs at Chromosome 3L (40 k resolution).

D Relative contact probability (RCP) plot showing corrected contact counts at different genomic ranges, genome widely. RCP values are compared between EGFP (control)-KD (blue) and Nxf2-KD (orange) OSCs.

E Differential interaction heatmap for 16–19 Mbp of chr3L upon Piwi-KD or Nxf2-KD shown in $\log_2$ fold change. piRNA target TE insertions are indicated at the bottom (green filled triangles). Comparison between Piwi-KD and Nxf2-KD supports that Piwi and Nxf2 regulate chromatin conformation at the piRNA target region in a similar way.

F Average TAD separation score at piRNA target TE insertions (left) and non-piRNA target TE insertions (right) for Control (EGFP, left)- or Nxf2 (right)-KD (upper). Heatmap showing changes in the TAD separation score for each piRNA target TE insertion sites (lower). This analysis indicates that a small decrease in separation score occurs upon Nxf2-KD at piRNA target TE insertion sites, as in the case of Piwi-KD.

G Venn diagram displaying the number of TAD boundaries detected using Control (EGFP)- or Nxf2-KD Hi-C interactions. As in the case of Piwi-KD, only a small portion of boundaries were TAD boundaries observed specifically for Nxf2-KD (33 Nxf2-specific to 201 common boundaries).

 © 2021 The Authors

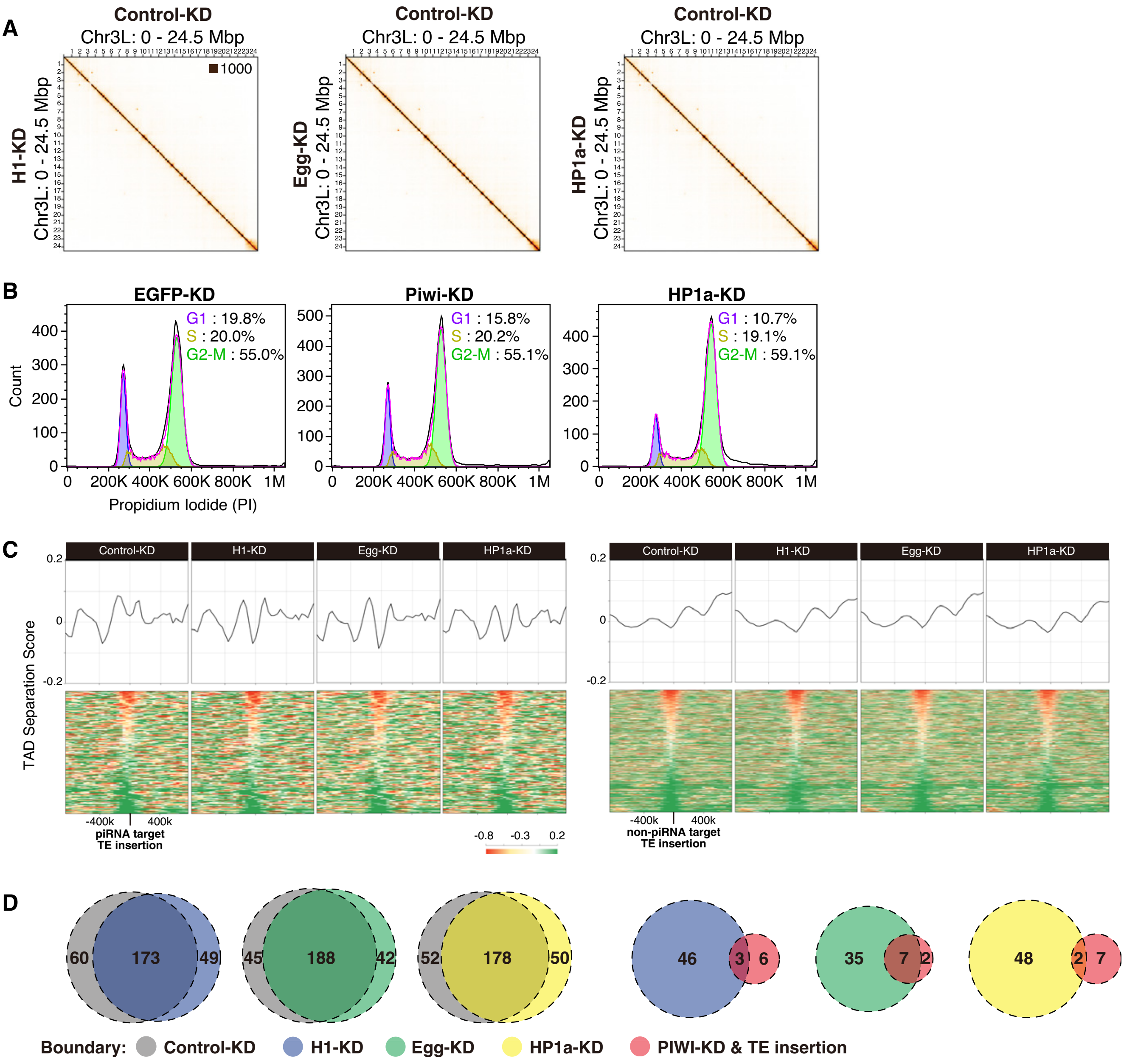

Figure EV5. Chromatin conformational changes induced by H1, Egg, and HP1a.

A Hi-C interaction matrices of the Control (EGFP)-KD and H1 (left)-, Egg (middle)-, or HP1a (right)-KD OSCs at Chromosome 3L (40 k resolution).

B Cell cycle analysis of OSCs with KD of EGFP (control), Piwi, or HP1a. Correlated dual-variable plots of cell counts versus propidium iodide uptake. The ratio of G1 (purple), S (yellow), and G2-M (green) cell populations is indicated. A decrease in the G1 population and an increase in the G2-M population are observed for HP1a-KD.

C Average TAD separation score at piRNA target TE insertions and non-piRNA target TE insertions for Control (EGFP)-, H1-, Egg-, or HP1a-KD (upper). Heatmap showing changes in the TAD separation score for each piRNA target TE insertion sites (lower). This analysis indicates that a weak decrease of separation score upon indicated KDs occurs at piRNA target TE insertion sites.

D Venn diagrams displaying the number of TAD boundaries detected using EGFP- and H1 (left)-, Egg (middle), or HP1a (right)-KD Hi-C interactions (left). Venn diagrams of TAD boundaries detected specifically for H1 (left)-, Egg (middle)-, or HP1a (right)-KD OSC, and TAD boundaries that are Piwi-KD specific and harbor piRNA target TE insertions (right). The number of boundaries observed specifically for H1 and HP1a-KD was relatively high (49 and 50). However, the overlap with Piwi-KD boundaries was low (3 and 7). In the case of Egg-KD, 7 boundaries out of 42 Egg-KD specific boundaries were shared with Piwi-KD specific boundaries.

© 2021 The Authors
