# Supplementary figures and images for: Piwi–piRNA complexes induce stepwise changes in nuclear architecture at target loci
Source: EMBO J. 2021 Aug 2;40(18):e108345. doi: 10.15252/embj.2021108345 (PMC8441340; doi:10.15252/embj.2021108345)

Figure 1C

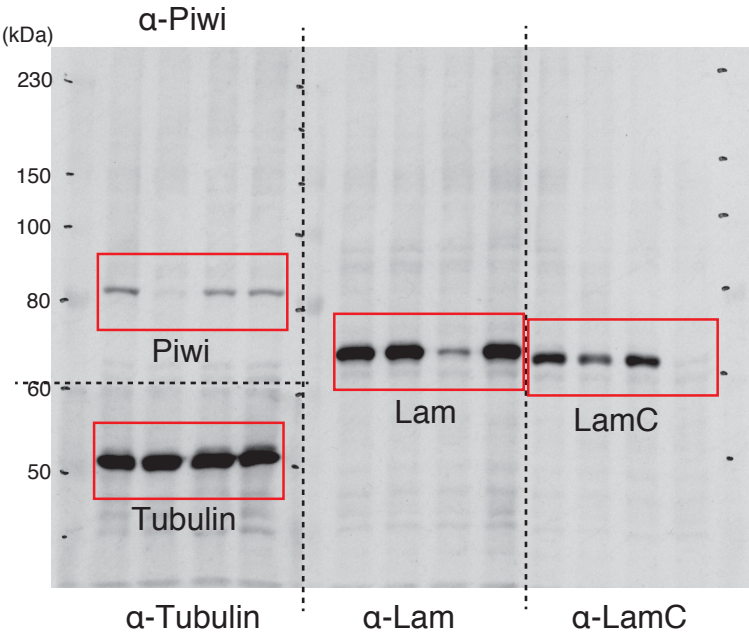

Supplement: Supplementary file 6 — Source Data for Figure 1 [file EMBJ-40-e108345-s005.pdf]

Figure 5A

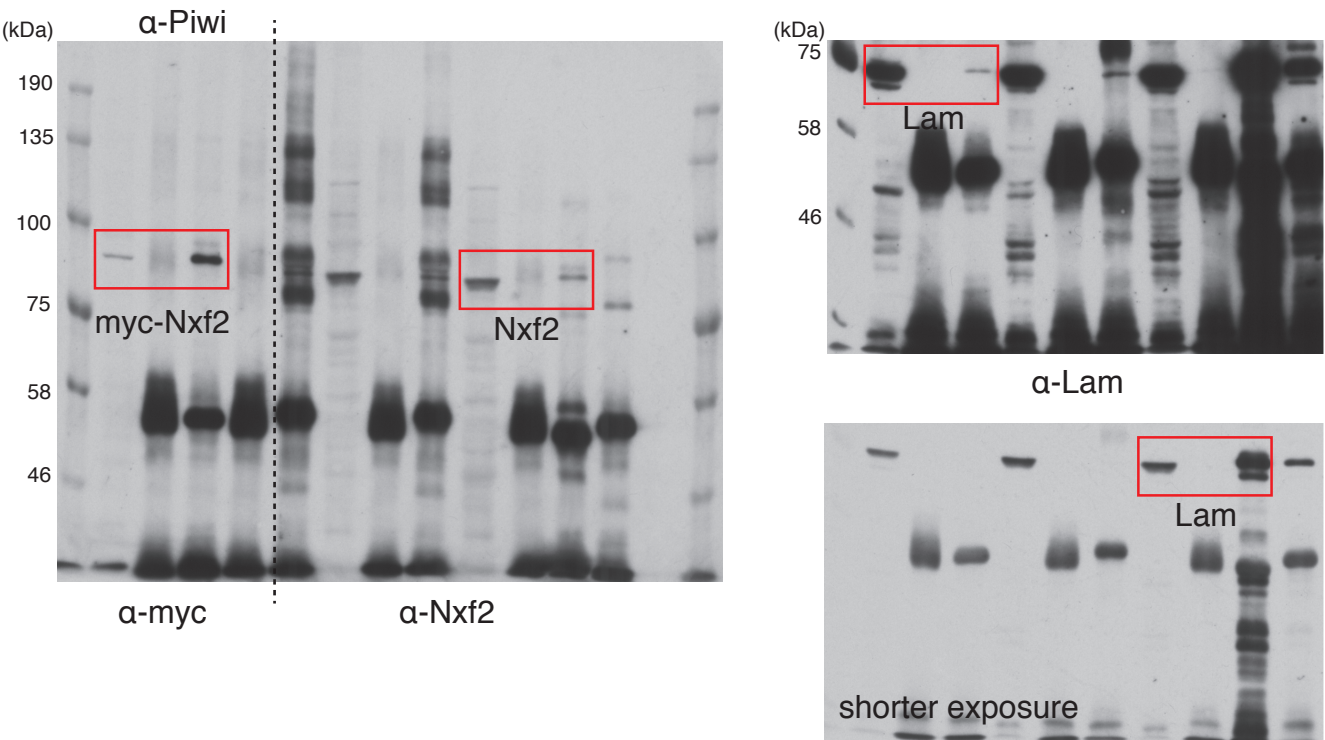

Supplement: Supplementary file 7 — Source Data for Figure 5 [file EMBJ-40-e108345-s004.pdf]
